# DNAJC24 is a potential therapeutic target in hepatocellular carcinoma through affecting ammonia metabolism

**DOI:** 10.1038/s41419-022-04953-z

**Published:** 2022-05-24

**Authors:** Guangtao Li, Yuchao He, Hui Liu, Dongming Liu, Lu Chen, Yi Luo, Liwei Chen, Lisha Qi, Yun Wang, Yingying Wang, Yu Wang, Linlin Zhan, Ning Zhang, Xiaolin Zhu, Tianqiang Song, Hua Guo

**Affiliations:** 1 Department of Tumor Cell Biology, Tianjin Medical University Cancer Institute and Hospital, Tianjin, 300060 China; 2 Department of Hepatobiliary Cancer, Liver Cancer Research Center, Tianjin Medical University Cancer Institute and Hospital, Tianjin, 300060 China; 3 National Clinical Research Center for Cancer, Key Laboratory of Cancer Prevention and Therapy, Tianjin’s Clinical Research Center for Cancer, Tianjin, 300060 China; 4 Department of Pathology, Tianjin Medical University Cancer Institute and Hospital, Tianjin, 300060 China

**Keywords:** Oncogenes, Prognostic markers, Macroautophagy

## Abstract

Evolutionarily conserved heat shock proteins are involved in the heat shock response of cells in response to changes in the external environment. In normal tissues, heat shock proteins can help cells survive in a rapidly changing environment. Likewise, in malignant tumors heat shock proteins may help tumor cells cope with external stresses as well as the stress of treatment. In this way they become accomplices of malignant tumors. Here we demonstrated for the first time that high expression of DNAJC24 (a heat shock protein) shortens survival in patients with HCC by immunohistochemical staining of 167 paired hepatocellular carcinomas and paraneoplastic tissues as well as data from public databases. In vitro experiments demonstrated that stimuli such as hypoxia, starvation and heat could upregulate DNAJC24 expression in HCC cells through transcriptional regulation of HSF2, and high expression of DNAJC24 in HCC cells could promote the proliferation and motility of HCC cells. In addition, we also verified that targeting DNAJC24 under normal culture conditions can affect the proliferation and autophagy of HCC cells by interfering with ammonia metabolism, thereby inhibiting the malignant progression of HCC. Overall, we suggested that DNAJC24 may become a new target for the treatment of HCC.

## Introduction

Primary liver cancer is the sixth most commonly diagnosed cancer and the third leading cause of cancer death worldwide [1]. Hepatocellular carcinoma (HCC) is the most common primary liver cancer, with a reported 5-year survival rate of 47–53% even in patients with early, small HCC (<3 cm) who undergo surgical resection [2, 3]. The poor prognosis of HCC remains a challenge due to high rates of metastasis and postoperative recurrence [4, 5]. Therefore, a deep understanding of the mechanisms underlying HCC progression and the development of new therapeutic strategies are urgently needed.

Cancer cells inevitably encounter stress during tumor development [6]. These stresses include hypoxia [6], starvation [7], thermal stress [8], acidosis [9] and the therapeutic interventions, among others. Heat shock proteins (HSPs) are a group of highly conserved proteins that are produced in response to natural or unnatural stresses [10]. They can prevent apoptosis induced by mitochondrial outer membrane permeabilization, cytochrome *c* release, apoptosome assembly, caspase activation and TNF death receptors [11]. DNAJC24 is a member of the type III DNAJ/HSP40 subfamily, which has the most member of all human HSP families. Members of this subfamily, DNAJA1 [12], DNAJB1 [13], DNAJB8 [14], DNAJC6 [15], and DNAJC12 [16], have been found to have important functions in cancer development or serve as diagnostic biomarkers. However, the role of DNAJC24 in malignancy has never been reported.

Therefore, in this study, we applied a combination of online data mining, high throughput “omics” technologies, biochemistry and molecular biology to investigate the role and underlying mechanisms of DNAJC24 in HCC.

## Materials and methods

### Patients and tissue specimens

A total of 167 pairs of cancerous and matched adjacent nonmalignant tissue samples were obtained from patients with hepatocellular carcinoma who underwent radical surgical resection between June 2010 and December 2014 at Tianjin Medical University Cancer Institute and Hospital (Tianjin, China). Patients who had nonradical local treatments and systemic treatments were excluded from the study. The relationship between clinicopathologic characteristics and DNAJC24 expression in the 167 patients is summarized in Table S1. Pathologists examined all paraffin-embedded specimens using hematoxylin and eosin staining to confirm that tumor tissue contains more than 70% tumor cells and non-tumor tissue doesn’t contain tumor cells. All patients in this study provided written informed consent for sample collection and data analyses. The study was consistent with the ethical guidelines of the Helsinki Declaration and approved by the Ethics Committee.

### Immunohistochemistry

Immunohistochemistry (IHC) staining for DNAJC24 was performed in 167 pairs of cancerous and matched adjacent nonmalignant tissue. IHC staining for CD31 was performed in the 166 cancerous tissues. The tissues were sequentially immersed in xylene and gradient ethanol to dewax and rehydration. Antigen retrieval was performed using citrate target retrieval solution. pH 6.0 (ZSGB-BIO, ZLI-9065) in a pressure cooker for 2.5 min then cooled naturally to room temperature. 3% hydrogen peroxide was used to inhibit endogenous hydrogen peroxidase activity. Subsequently, the samples were incubated with rabbit anti-DNAJC24(1:50, Abcam, ab246925) and rabbit anti-CD31(1:50, Abcam, ab28364) at room temperature for 30 min and overnight at 4 °C. After washed with phosphate-buffered saline (PBS), the samples were stained with secondary antibody for 1 h at room temperature. The cells were visualized with 3,3-diaminobenzidine solution (ZSGB-Bio, ZLI-9017) and counterstained with hematoxylin. The final IHC score of DNAJC24 was the product of the staining intensity score and percentage score. A final staining score ≥1 was defined as DNAJC24 high expression; the final staining score <1 was defined as DNAJC24 low expression. The staining intensity score of DNAJC24 was evaluated in four classes: 0, no immune response; 1, weak immune response; 2, medium strong immune response; 3, strong immune response. The staining percentage score of DNAJC24 was classified on a 4-point scale: 0, no positive cells; 1, <30% positive cells; 2, 30–60% positive cells; and 3, 60–100% positive cells. The density of CD31 < 30% was defined as CD31-Low, while ≥30% was defined as CD31-High.The sections were photographed using a light microscope (Olympus BX61).

### Cell culture

PLC and HEK293T cells were purchased from American Type Culture Collection (ATCC; Manassas, VA, USA). Huh7 cells were purchased from the Cell Bank of the Chinese Academy of Sciences (Shanghai, China). The cell lines were cultivated in DMEM medium (Corning, NY, USA), supplemented with 1% penicillin/streptomycin (HyClone, Logan, UT, USA) and 10% fetal bovine serum (PAN-Seratech, Edenbach, Germany) under culture requirements (37 °C; 5% CO_2_).

### Cell transfection

DNAJC24 shRNA was used to knockdown this gene. Packaging plasmids (VSVG and ΔR) and expression plasmids (OE and matched Ctrl; KD and matched Ctrl) were transfected into HEK293T cells using Lipofectamine 2000 (Invitrogen). After 48 h, the medium supernatant was collected to obtain lentiviral particles. PLC and Huh7 cells were infected with lentivirus to produce stable DNAJC24 knockdown (KD), DNAJC24 overexpression (OE) and matched control (Ctrl) cell lines. The sequences of DNAJC24 shRNA were listed in Table S2.

### Western blotting and antibodies

Western blotting was conducted as described previously [17]. The following antibodies were used: anti-DNAJC24 (1:500) from Abcam, anti-β-actin (1:1000) from Santa Cruz Biotechnology, anti-LC3B (1:1000) from Cell Signaling Technology, anti- p62 (1:1000) from Santa Cruz Biotechnology, anti-HSF2 (1:500) from ABclonal, anti-CPS1(1:500) from ABclonal, anti-Caspase3 (1:1000) from Cell Signaling Technology, anti-Caspase9 (1:1000) from Cell Signaling Technology, goat anti-mouse IgG-HRP from Santa Cruz Biotechnology, and goat anti-rabbit IgG-HRP from Santa Cruz Biotechnology.

### Quantitative real-time PCR (qRT-PCR)

Total RNA was extracted from cells using TRIzol reagent (Ambion). cDNA was synthesized by RNA reverse transcription using the PrimeScript™ RT Master Mix kit (Takara, Japan). The amplification reaction was performed using the AceQ qPCR SYBR Green Master Mix kit according to the manufacturer’s instructions (Vazyme, China). Primer sequences are listed in Table S3.

### Cell viability assay, colony formation assay, chemotaxis assay, invasion assay

Cell viability assays, colony formation assays, chemotaxis assays, invasion assays were performed as described previously [17]. According to the manufacturer’s instructions, cell proliferation assay was performed using the BeyoClick™ EdU-594 Cell Proliferation Kit (Beyotime Biotech. Inc.).

### Immunofluorescence staining

Cells were seeded in twelve-well plates, fixed with 4% paraformaldehyde for 30 min at room temperature, washed with PBS and permeabilized with 0.2% Triton X-100 in PBS for 15 min. Cells were then blocked with 10% normal donkey serum for 60 min at room temperature. Subsequently, the cells were incubated overnight at 4 °C with rabbit anti-Ki67 (0.5 µg/ml, Abcam, ab15580). Then, the cells were incubated with Alexa-Fluor-conjugated secondary antibodies (Invitrogen) in 1% BSA for 1 h at room temperature. DAPI was used to counterstain the nuclei, and images were obtained by a fluorescence microscope (Invitrogen, EVOS M5000).

### Dual-luciferase reporter assays

The transcriptional effect of HSF2 on DNAJC24 was assessed by dual-luciferase assay. The promoter region of DNAJC24 was amplified and the predicted binding sites -TTTTGGAACGTTT-was mutated to -GGGGTTCCATGGG-. Then, the wild type promoter and mutant type promoter of DNAJC24 were ligated into the luciferase reporter plasmid pPRO-RB-Report (RiboBio, China). The effect of HSF2 on DNAJC24 promoter activity was measured using a dual luciferase assay kit (Promega, E2920).

### Protein synthesis assay

Global protein synthesis was assayed using a protein synthesis assay kit (Cayman Chemical,601100) according to the manufacturer’s instructions. Briefly, cells were seeded in 96-well plates and incubated for 30 min with o-propargyl-puromycin working solution in complete medium. Subsequently, cells were fixed with cell-based assay fixative, stained with 5 FAM-azide solutions, examined by a fluorescent plate reader (Bio-Tek Synergy H1) using a filter designed to detect FITC (excitation/emission = 485/535 nm) and photographed under a fluorescence microscope.

### Assessment of intracellular ammonia

An ammonia assay kit (Abnova, KA0810) was used to detect intracellular ammonia according to the manufacturer’s instructions. Briefly, a total of 2 × 10^6^ cells were washed with PBS and lysed in 100 µL assay buffer provided in the kit. A BCA protein assay kit (Thermo,23227) was used to assay the cell lysate protein concentration. Add 30 µg of cell lysate to 96 well plates, bring the volume to 50 µL/well with assay buffer. Add 50 µL of the Reaction Mix (42 µL Ammonia Assay Buffer, 2 µL Oxi red probe, 2 µL Enzyme Mix, 2 µL Developer, and 2 µL Converting Enzyme) to each well containing the test samples. Add 50 µL Sample Control Mix (44 µL Ammonia Assay Buffer, 2 µL Oxi red probe, 2 µL Enzyme Mix, and 2 µL Developer) to Sample Control. Mix well. The reaction was incubated for 1 h at 37 °C, protect from light. Optical density was measured at 570 nm using a microplate reader (BioTek Synergy H1).

### Measurement of autophagic flux

Cells were transfected with the stubRFP-sensGFP-LC3 lentivirus purchased from GENE-CHEM following the manufacturer’s protocol. Then, cells were visualized using laser scanning confocal microscopy (LSM800, Zeiss). Autophagosomes were labeled yellow (mRFP and GFP) whereas autolysosomes were labeled red (mRFP only).

### Bioinformatics analysis

We downloaded the raw data of HCC from TCGA (National Cancer Institute) official website. Then, the data were normalized by R Studio. We analyzed the expression of DNAJC24 and HSF2 using R Studio. The GEPIA (Gene Expression Profiling Interactive Analysis, cancer-pku.cn) online database or R Studio was used to analyze the correlation. Correlations between DNAJC24, HSF2 mRNA level and survival in patients with HCC were analyzed using the GEPIA online database. We used the online program JASPAR (http://jaspar.genereg.net/analysis) to search for potential transcription factors with binding sites in the DNAJC24 promoter.

### Statistical analysis

Statistical analyses were performed using SPSS 25.0 or GraphPad Prism 8.0. All data are shown as the means ± SD. Differences between groups were evaluated by a two-tailed Student’s *t* test or one-way ANOVA. The univariate Kaplan–Meier method and multivariate Cox method were used to analyze the independent risk factors and survival curve of HCC patients. Statistical significance was defined as *P* < 0.05.

## Results

### DNAJC24 expression is elevated in HCC tissues and correlates with poor prognosis

Previously, our group found that cPLA2α [18, 19] and PNO1 [20] play a key role in the malignant progression of HCC. Interestingly, we examined the whole transcriptome of cells with different levels of cPLA2α and PNO1 and found that with the decrease of cPLA2α and PNO1, the expression of DNAJC24, a heat shock protein, also decreased (Fig. S1A, B). In addition, we found that DNAJC24 was positively correlated with cPLA2α and PNO1 at the mRNA level in HCC (Fig. S1C, D). These results made us interested in the role of DNAJC24 in HCC progression.

**Fig. 1:**
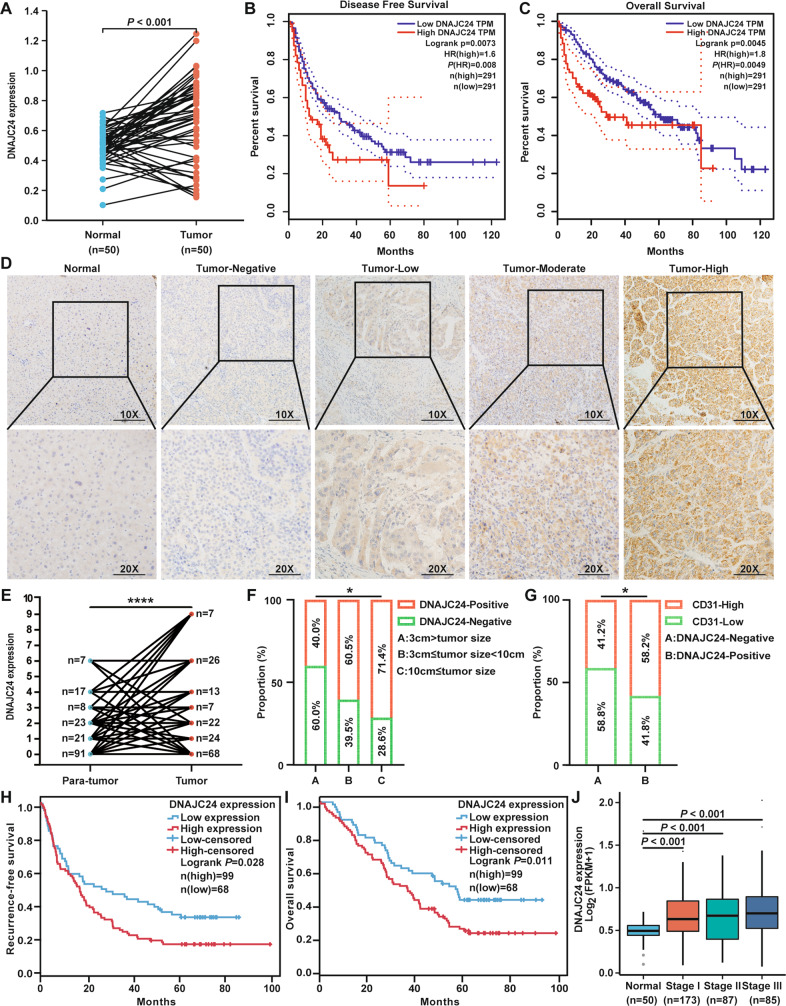
DNAJC24 expression is generally upregulated in human HCC tissues and is associated with poor patient prognosis. **A** Expression of DNAJC24 in cancer and paired normal liver tissues of 50 HCC patients based on data from the TCGA hepatocellular carcinoma dataset. **B**, **C** The Kaplan–Meier survival analysis of disease-free survival (**B**) and overall survival (**C**) of HCC patients analyzed by GEPIA (top 20%, high; bottom 80%, low). **D** Representative images of DNAJC24 in 167 HCC and paired para-tumor tissues stained with IHC (scale bar,200 μm or 100 μm). **E** IHC analysis of DNAJC24 expression in 167 pairs tissue of HCC specimens. **F** Positive rate of DNAJC24 in different tumor size subgroups (Group A, 25 cases; Group B, 114 cases; Group C 28 cases). **G** Percentage of high and low microvessel density in the DNAJC24 positive and negative subgroups. **H**, **I** The Kaplan–Meier survival analysis of recurrence-free survival (**H**) and overall survival (**I**) in 167 HCC patients. **J** Box plots of DNAJC24 expression in normal liver and tumor tissues (Clinical stages I to III) from the TCGA Database. *P < 0.05, ****P < 0.0001.

To evaluate the role of DNAJC24 in the progression of HCC, we first analyzed that DNAJC24 mRNA expression was significantly upregulated in tumors compared with normal tissues based the TCGA Liver Hepatocellular Carcinoma dataset (*n* = 50) (Fig. 1A). Moreover, DNAJC24 mRNA expression was higher in tumors with more advanced stage (Fig. 1J). Data from the GEPIA online database showed that patients with high DNAJC24 mRNA expression had a worse prognosis, both in terms of disease-free survival and overall survival (OS) (Fig. 1B, C).

Next, we examined the protein expression of DNAJC24 in 167 paired HCC and non-tumor samples by IHC assays to further identify the effect of DNAJC24 on the prognosis of HCC (Fig. 1D). The relationship between clinicopathologic characteristics and DNAJC24 expression in the 167 patients is summarized in Table S1. The protein expression of DNAJC24 were also significantly higher in tumor than in corresponding non-tumor tissues (Fig. 1E). The positive rate of DNAJC24 increased with increasing tumor diameter (Fig. 1F). In HCC, tumor progression is associated with angiogenesis and microvascular density is associated with poor prognosis [21]. Therefore, we measured endothelial vessel density by anti-CD31 IHC. Interestingly, we found a higher density of CD31 in DNAJC24-positive tumors (Fig. 1G), suggesting that microvessel density in HCC was associated with DNAJC24 expression levels. We further explored the relationship between the protein expression of DNAJC24 and HCC patients’ survival. Based on DNAJC24 protein expression levels, patients were divided into the DNAJC24 high expression group (IHC staining positive, *n* = 99) and the low expression group (IHC staining negative, *n* = 68). Kaplan–Meier survival curves suggested that HCC patients with higher DNAJC24 protein expression had worse recurrence-free survival (RFS) and OS (Fig. 1H, I). There are gender differences in the incidence and prognosis of HCC [22–24]. Therefore, we did survival analysis after grouping 167 patients by gender. The results for the male group were consistent with the total population (Fig. S2A, C), and there was a trend for the female group to be consistent with the total population (Fig. S2B, D). The Cox regression model showed that increased DNAJC24 expression was an independent risk factor for the OS and RFS of HCC patients (Table 1). Then, we compared the accuracy of predictive survival probability by two nomogram models (with or without DNAJC24 staining score) using the C-index (Fig. S3). The C-index of the model with the DNAJC24 staining score was higher than that without the DNAJC24 staining score (0.661 vs. 0.636), suggesting that adding DNAJC24 staining score into the nomogram model increases the predictive value (Fig. S3). Taken together, by using IHC staining of a large number of HCC tissues and online database, we found that DNAJC24 expression was elevated in HCC tissues and was an independent risk factor for HCC prognosis, while DNAJC24 expression levels correlated with tumor size and microvessel density.

**Table 1:** Univariate and multivariate analysis of prognostic factors associated with OS and DFS in 167 HCC patients.

HCC patients (*n* = 167)	Number	Univariate analysis		Multivariate analysis		Univariate analysis		Multivariate analysis	
		5-year OS (%)	*P* value	HR (95% CI)	*P* value	5-year DFS (%)	*P* value	HR (95% CI)	*P* value
Age(years): ≥55/<55	92/75	38.6/42.0	0.742			18.0/17.5	0.895		
Sex: male/female	137/30	41.5/28.4	0.097			18.8/15.0	0.201		
HBV: Yes/No	131/36	36.9/53.2	0.226			17.0/34.2	0.224		
Liver cirrhosis: Yes/No	90/77	41.5/38.6	0.741			17.0/18.9	0.322		
Tumor size(cm): ≥3/<3	142/25	38.6/49.4	0.12			15.7/37.4	0.055		
Mavi: Yes/No	15/152	14.9/42.1	**0.008**	1.128 (0.482, 2.623)	0.780	4.9/19.7	**0.000**	2.062 (0.904, 4.703)	0.085
Mivi: Yes/No	93/74	29.3/49.4	**0.025**	1.495 (1.000, 2.235)	**0.050**	12.0/21.2	0.101		
AFP(ng/ml): ≥20/<20	86/81	29.0/51.5	**0.004**	1.308 (0.933, 1.832)	**0.004**	11.9/26.0	**0.002**	1.000 (1.000, 1.000)	**0.0001**
BCLC stage: 0&A/B&C	141/26	46.7/15.9	**0.001**	1.963 (1.007, 3.825)	**0.048**	19.8/6.538	**0.000**	1.535 (0.794, 2.965)	0.202
Staining score of DNAJC24: ≥1/<1	99/68	36.5/54.2	**0.011**	2.048 (1.352, 3.101)	**0.001**	16.8/26.4	**0.028**	1.643 (1.129, 2.391)	**0.009**

Bold is used to emphasize that the value is statistically significant.

*AFP* α-fetoprotein, *BCLC* Barcelona Clinic Liver Cancer, *HBV*, hepatitis B virus, *Mavi* macrovascular invasion, *Mivi* microvascular invasion.

### DNAJC24 promotes the proliferation and motility of HCC cells

**Fig. 2:**
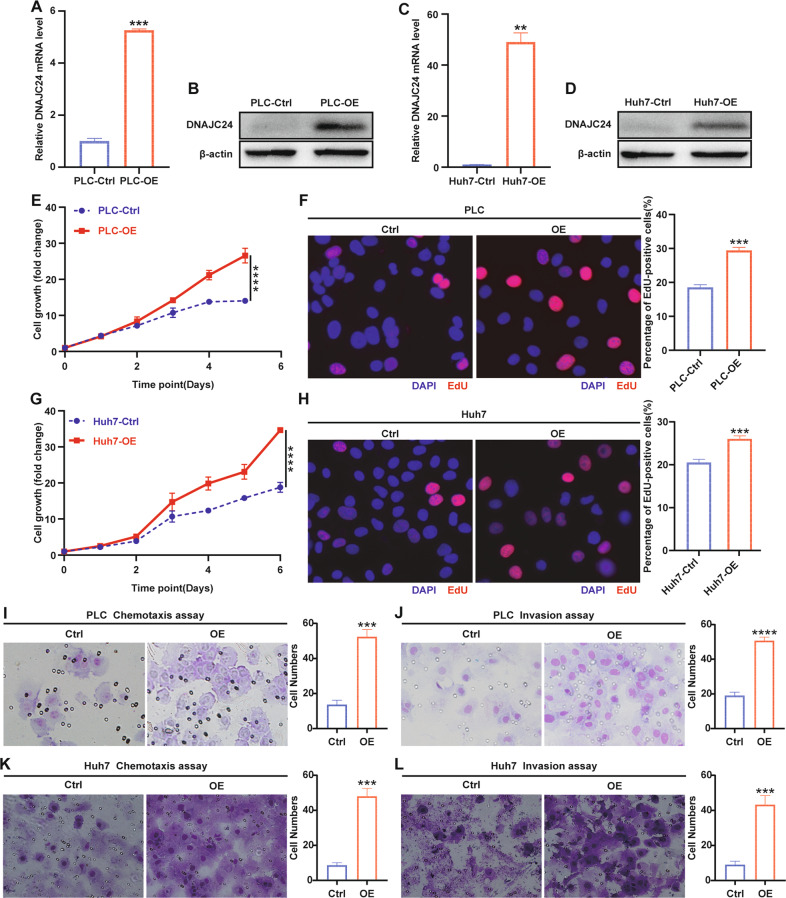
DNAJC24 promotes proliferation and motility of HCC cells in vitro. **A**, **C** PLC and Huh7 cells were infected with a lentivirus to produce stable DNAJC24 overexpression (OE) cells. qRT-PCR was performed to determine levels of DNAJC24 mRNA. β-actin was used as an internal control. **B**, **D** Western blotting was performed to determine levels of DNAJC24 protein. **E**, **G** CCK-8 cell viability assay analysis of the impact of DNAJC24 ectopic overexpression on PLC (**E**) and Huh7 (**G**) cell growth. Results were normalized to viability at day 0 and represented as fold change. **F**, **H** The effect of ectopic overexpression of DNAJC24 on the proliferation of PLC (**F**) and Huh7 (**H**) cells was analyzed by EdU staining. Representative images and EdU positive cell rates are as shown. **I**–**L** Chemotaxis (**I**, **K**) and Matrigel invasion (**J**, **L**) assays were used to detect the effect of ectopic overexpression of DNAJC24 on PLC (**I**, **J**) and Huh7 (**K**, **L**) motility. Data were presented as mean ± SEM. *n* = 2–5. **P < 0.01, ***P < 0.001, ****P < 0.0001.

Then, we investigated the biological functions of DNAJC24 in HCC cells. Full-length human DNAJC24 was cloned into a lentiviral vector and then stably transfected into the PLC and Huh7 human HCC cell lines. The overexpression efficiency of DNAJC24 was confirmed by qRT-PCR and Western blotting (Fig. 2A–D). DNAJC24 overexpression resulted in a considerable promotion effect on the proliferation of HCC cells as evidenced by the CCK-8 cell viability assay (Fig. 2E, G). EdU staining assays also confirmed that ectopic overexpression of DNAJC24 promoted the proliferation of HCC cells (Figs. 2F, H). In vitro chemotaxis and Matrigel invasion assays indicated that DNAJC24 overexpression efficiently increased the motility of PLC (Fig. 2I, J) and Huh7 (Fig. 2K, L) cells. In summary, we verified that overexpression of DNAJC24 can promote the proliferation and motility of HCC cells, which in turn could lead to poor prognosis of HCC patients.

### Under stress, heat shock factors 2 upregulates DNAJC24 expression in HCC cells

**Fig. 3:**
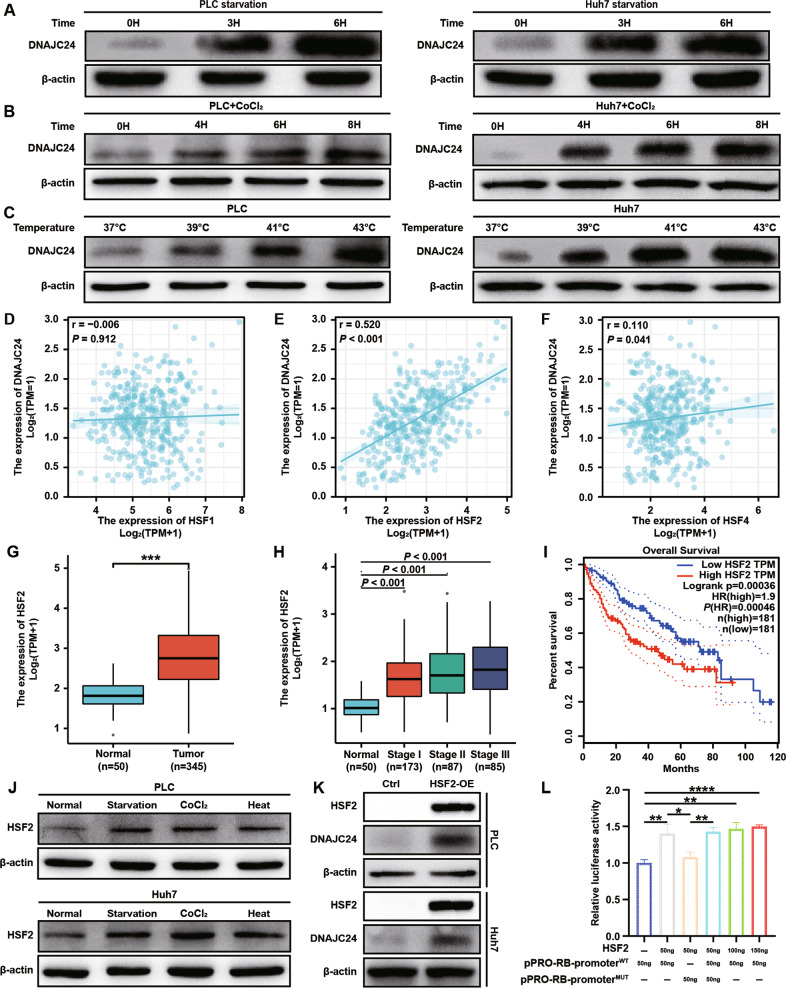
External stimuli such as starvation, hypoxia and heat upregulate DNAJC24 expression through HSF2 in HCC cells. **A** The cell culture medium of PLC and Huh7 was changed to PBS and the cells were cultured in PBS for 0h, 3h and 6h respectively and then the cells were lysed. Western blotting was performed to determine levels of DNAJC24 protein. **B** Adding CoCl_2_ (final concentration 200 μM) to the medium of PLC and Huh7 cells to simulate hypoxic environment, and the cells were incubated in it for 0h, 4h, 6h, 8h respectively and then lysed. Western blotting was performed to determine levels of DNAJC24 protein. **C** PLC and Huh7 cells were cultured at 37 °C, 39 °C, 41 °C, 43 °C respectively in 5% CO_2_ for 3h, and then the cells were lysed. Western blotting was performed to determine levels of DNAJC24 protein. **D**–**F** Analysis of the possible correlation between DNAJC24 and HSF1 (**D**), HSF2 (**E**), HSF4 (**F**) mRNA levels based on the data from the TCGA HCC dataset. Data were analyzed using Spearman’s rank correlation coefficient. **G** Box plots of HSF2 expression in cancer and paired normal liver tissues of HCC patients based on data from the TCGA HCC dataset. **H** Box plots of HSF2 expression in normal liver and tumor tissues (Clinical stages I–III) based on data from the TCGA HCC database. **I** The Kaplan–Meier survival analysis of overall survival of HCC patients analyzed by GEPIA (top 50%, high; bottom 50%, low). **J** Lysis of PLC and Huh7 cells after applying starvation, hypoxia, and heat (40 °C) stimulation to 3h. Western blotting was performed to determine levels of HSF2 protein. **K** Stable cell lines with HSF2 overexpression were constructed using lentiviral transfection in PLC and Huh7 cells, and the protein levels of HSF2 and DNAJC24 were detected by Western blotting after cell lysed. **L** DNAJC24 wild-type promoter (pPRO-RB-promoter^WT^) and mutant promoter (pPRO-RB-promoter^MUT^)were ligated to the reporter vector. A dual luciferase assay was performed to assess the effects of HSF2 overexpression on DNAJC24 transcription in PLC cells. Data were presented as mean ± SEM. *n* = 3. *P < 0.05, **P < 0.01, ***P < 0.001, ****P < 0.0001.

HSPs are essential components of a cell’s defense mechanism against stress injuries [25]. Of all human HSP families, the DNAJ/HSP40 family has the largest number of members. All of the DNAJ/HSP40s contain a J domain through which they bind to Hsp70s and can be categorized into three subfamilies (DNAJA, DNAJB, DNAJC) [26]. DNAJC24 belongs to type III DNAJ/HSP40s which have only one J domain. Since HSPs are usually responsive to environmental stress, we wanted to explore whether DNAJC24 is responsive to external stressful stimuli. We artificially upregulated the ambient temperature of PLC and Huh7 cells, and then detected changes in DNAJC24 expression by Western blotting and qRT-PCR. As expected, as the ambient temperature increased, the expression of DNAJC24 was upregulated and showed a certain degree of stimulus intensity dependence (Figs. 3C and S4C). In addition, we changed the medium to PBS to simulate starvation stimulation or added CoCl_2_ (200 μM) to the medium to simulate hypoxia. Interestingly, HCC cells upregulated DNAJC24 expression after stimulation by starvation and hypoxia and the extent of DNAJC24 upregulation showed some time dependence (Figs. 3A, B and S4A, B). These results showed that the expression of DNAJC24 in HCC cells increased after being stimulated by the external stressful stimuli.

The HSFs (heat shock transcription factors) were direct transcriptional factors of some genes encoding HSPs [27]. However, whether HSFs can regulate DNAJC24 expression or which member regulates DNAJC24 expression hasn’t been reported. Among vertebrates, HSF 1, 2, and 4 are ubiquitous, whereas HSF3 has been characterized only in avian species [28]. The correlation analysis using TCGA database showed that DNAJC24 expression was highly positively correlated with HSF2 expression (Fig. 3E), but weakly correlated with HSF1 (Fig. 3D) and HSF4 (Fig. 3F). HSF2 expression was also higher in HCC tissues than in para-cancerous tissues (Fig. 3G), and the HSF2 expression level increased with tumor stage (Fig. 3H). These are consistent with the expression of DNAJC24 in HCC tissues. As above, we detected changes in HSF2 expression in HCC cells after being stimulated by starvation, hypoxia, and heat. Similarly, HSF2 expression levels were upregulated with the administration of these stress stimuli (Fig. 3J). More importantly, as with DNAJC24, OS was shorter in patients with high HSF2 expression (Fig. 3I). In addition, we constructed HSF2 overexpression cell lines in PLC and Huh7 cells (Figs. S5A, B and 3K) and found that DNAJC24 expression was upregulated in HSF2-OE cells (Figs. S5C, D and 3K).

Next, we searched the JASPAR website and found that HSF2 may bind to a segment of the DNAJC24 promoter sequence (5’-TTTTGGAACGTTT-3’), thereby regulating DNJAC24 expression (Fig. S5E). We amplified the core promoter region of DNAJC24 and mutated 5’-TTTTGGAACGTTT-3’ to 5’-GGGGTTCCATGGG-3’. Then, the wild type promoter and mutant type promoter of DNAJC24 were ligated into pPRO-RB-Report. Subsequent dual-luciferase reporting experiments shown that HSF2 could transcriptionally regulate DNJC24 expression, and mutating the predicted binding site could inhibit HSF2 transcriptional regulation of DNAJC24 (Fig. 3L). These data suggest that HSF2 is a transcriptional regulator of DNAJC24.

### Knockdown of DNAJC24 inhibits proliferation, motility, and protein synthesis in HCC cells

**Fig. 4:**
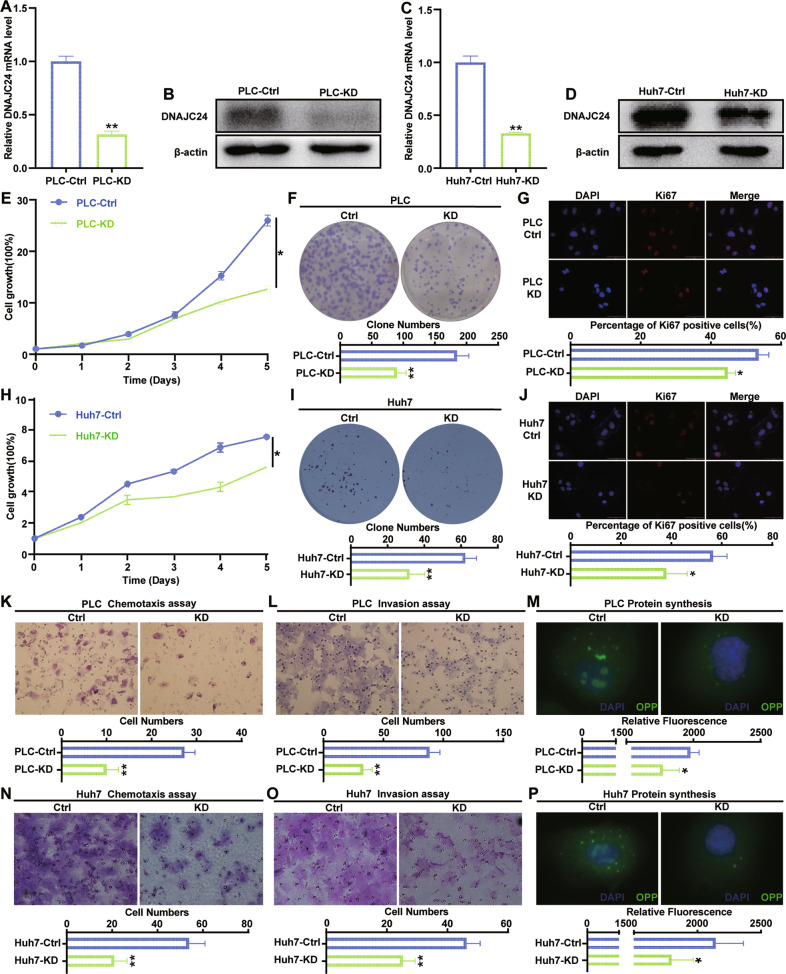
Knockdown of DNAJC24 inhibits proliferation, motility, and protein synthesis in HCC cells in vitro. **A**, **C** PLC and Huh7 cells were infected with a lentivirus to produce stable DNAJC24 knockdown (KD) cells. qRT-PCR was performed to determine levels of DNAJC24 mRNA. β-actin was used as an internal control. **B**, **D** Western blotting was performed to determine levels of DNAJC24 protein. **E**, **H** CCK-8 cell viability assay analysis of the impact of DNAJC24 knockdown on PLC (**E**) and Huh7 (**H**) cell growth. Results were normalized to viability at day 0 and represented as fold change. **F**, **I** Colony formation assay showing the effects of DNAJC24 knockdown on PLC (**F**) and Huh7 (**I**) cell growth. **G**, **J** Immunofluorescence staining detected Ki67 expression in DNAJC24-KD cells and control cells. Representative images and Ki67 positive cell rates are as shown. **K**, **L**, **N**, **O** Chemotaxis (**K**, **N**) and Matrigel invasion (**L**, **O**) assays were used to detect the effect of DNAJC24 knockdown on PLC (**K**, **L**) and Huh7 (**N**, **O**) motility. **M**, **P** Newly synthesized protein was detected in PLC (**M**) and Huh7 (**P**) cells after DNAJC24 knockdown using a protein synthesis assay kit. Data were presented as mean ± SEM. *n* = 2–3. *P < 0.05, **P < 0.01.

The above results suggest that environmental stress can upregulate DNAJC24 expression through HSF2, and upregulated DNAJC24 can promote the proliferation and motility of HCC cells, which in turn leads to poor prognosis of HCC patients. Does targeting DNAJC24 improve the poor prognosis of HCC patients? To answer this question, we constructed cell lines with stable down-expression of DNAJC24 in PLC and Huh7 cells by using the lentiviral vector carrying DNAJC24 shRNA. The efficiency of DNAJC24 knockdown was confirmed by qRT-PCR and Western blotting (Fig. 4A–D). DNAJC24 knockdown resulted in a considerable inhibitory effect on the proliferation (Fig. 4E, H) and colony-forming ability (Fig. 4F, I) of HCC cells. We found that the positive rate of Ki67 (a marker of cell proliferation [29]) was lower in DNAJC24-KD cells (Fig. 4G, J). This also supports that down-regulation of DNAJC24 can inhibit the proliferation of HCC cells. Migration and invasion assays verified that the down-regulation of DNAJC24 expression suppressed the migration and invasion abilities of PLC and Huh7 cells, respectively (Fig. 4K, L, N, O).

DNAJC24, also known as DPH4, is a homolog of the *S. cerevisiae* diphthamide methyltransferase proteins (DPHs). These enzymes are involved in the synthesis of diphthamide, and previous studies have shown that disrupting the expression of DPHs reduces protein synthesis [30, 31]. Therefore, we examined the effect of knocking down DNAJC24 on global protein synthesis and found that DNAJC24 knockdown could also inhibit protein synthesis (Fig. 4M, P). All these results suggest that DNAJC24 may be a potential therapeutic target for HCC.

### Knocking down DNAJC24 disrupts autophagic flow

Although the positive rate of Ki67 decreased in DNAJC24-KD cells, flow cytometric analysis of cell cycle revealed no increase in cells in G0 + G1 phase and no statistically significant differences in other phases (Fig. S6A), suggesting that DNAJC24 didn’t affect proliferation primarily by influencing the cell cycle. In addition, there was no significant effect of DNAJC24 knockdown on apoptosis by flow cytometric (Fig. S6B). The detection of apoptosis markers caspas3, caspase9 and their cleaved forms also didn’t reveal an increase in apoptosis in DNAJC24-KD cells (Fig. S6C, D). However, we did observe reduced growth in cells with DNAJC24 knockdown. Autophagy is an evolutionarily conserved process. It may promote the survival of tumor cells under the metabolic stress [32], but overactivated autophagy leads to another type of cell death [33]. Therefore, we investigated whether the reduction in cell growth induced by DNAJC24 knockdown is due to autophagy.

**Fig. 5:**
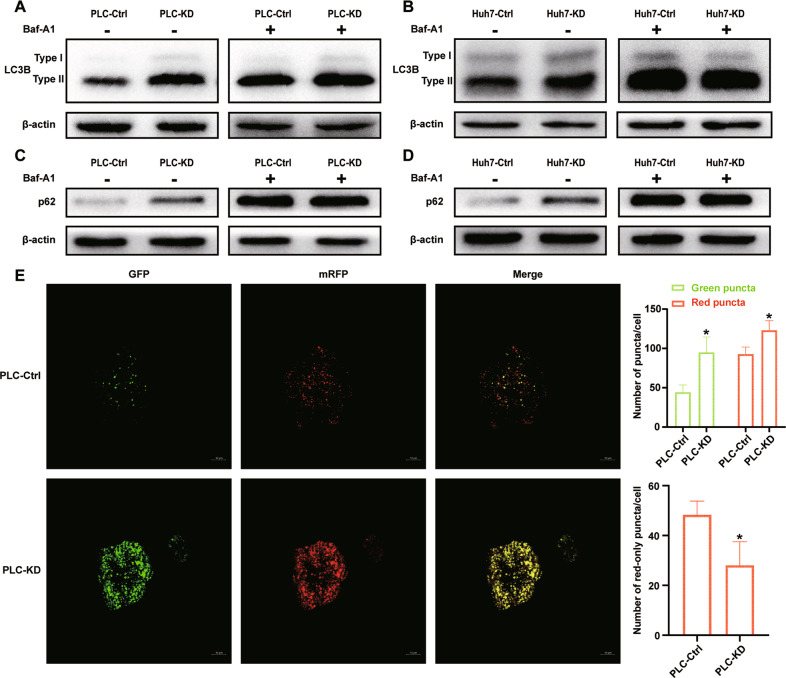
Knocking down DNAJC24 disrupts autophagic flow. **A**, **B**, **C**, **D** DNAJC24-KD cells and control cells were cultured under normal conditions or in medium supplemented with Bafilomycin A1 (final concentration 400 nM) for 24h. Western blotting was performed to determine protein levels of LC3B (**A**, **B**) and p62 (**C**, **D**) in cell lysates. **C** PLC DNAJC24-KD cells and control cells transfected with GFP-mRFP-LC3 lentivirus and analyzed by confocal microscopy. Quantification of the number of fluorescent puncta exhibiting green (GFP), red (mRFP) or red-only fluorescence (obtained by subtracting green puncta to the total red puncta) is shown (right graphs). Pictures at higher magnification are shown. Bars, 10 μm. Data were presented as mean ± SEM. *n* = 3. *P < 0.05.

First, we examined the changes of autophagy marker LC3B in DNAJC24-KD cells. LC3B-II was increased in DNAJC24-KD HCC cells (Fig. 5A, B left). Second, we detected changes in p62, which is a substrate for autophagy [34]. Surprisingly, we did observe a modest accumulation of p62 in DNAJC24-KD cells (Fig. 5C left and D left). In general, increased intracellular LC3B-II represents enhanced autophagy, and p62 should be reduced due to increased degradation. However, we observed a simultaneous increase in intracellular LC3B and p62. Considering that autophagy is a dynamic process including autophagic flux from of autophagosome to lysosome [34]. Thus the observed phenomenon may be caused by blocked fusion of autophagosome and lysosome or disrupted lysosome function, as previously reported [35, 36]. Next, we performed the experiments in the presence of the lysosomal pump inhibitor Bafilomycin A1(Baf-A1), which allows one to infer the rate of LC3-II neosynthesis [34, 35]. Interestingly, in the presence of Baf-A1, knocking down DNAJC24 didn’t result in the accumulation of LC3B-II (Fig. 5A right and B right), nor did p62 (Fig. 5C right and D right) change significantly in DNAJC24-KD cells, suggesting that DNAJC24 knockdown didn’t affect the initiation of autophagy. These experimental results all suggest that DNAJC24 knockdown affects the autophagolysosome phase of the autophagic flux.

To better assess the autophagic flux, PLCDNAJC24-KD and control cells were transfected with the stubRFP-sensGFP-LC3 lentivirus, which allows us to discriminate between early autophagosomes from acidified autophagolysosomes [37]. Consistent with the results obtained from Western blotting, we observed an increased number of mRFP-positive puncta and larger LC3 dots in DNAJC24-KD cells, suggesting more LC3 accumulation in DNAJC24-KD cells. Strikingly, by quantifying the number of red-only puncta (autophagolysosomes), we found that it was higher in control cells than in DNAJC24-KD cells (Fig. 5E). Thus, in DNAJC24-KD cells, the number of autophagosomes increased, and this increase wasn’t reflected in an equivalent increase in their maturation. In summary, we demonstrated that down-regulated expression of DNAJC24 interfered with the degradation of autophagosomes leading to the accumulation of LC3B and p62.

In addition, LC3B-II was increased and p62 was decreased in DNAJC24-OE HCC cells (Fig. S7A). This suggests that DNAJC24 overexpression enhanced autophagy in HCC cells. In the presence of Baf-A1, DNAJC24 overexpression resulted in a significant accumulation of LC3B-II and p62 (Fig. S7B), indicating that overexpression of DNAJC24 did promote the initiation of autophagy. Early-stage autophagy inhibitor 3-methyladenine (3MA) and late-stage autophagy inhibitor Baf-A1 could significantly inhibit the proliferation of PLC-DNAJC24-OE cells (Fig. S7C). These data suggest that autophagic flux increases when DNAJC24 is overexpressed and that blocking autophagy reduces the proliferation of DNAJC24-OE HCC cells.

### DNAJC24 knockdown inhibits HCC cells autophagy and proliferation by affecting the metabolism of ammonia

**Fig. 6:**
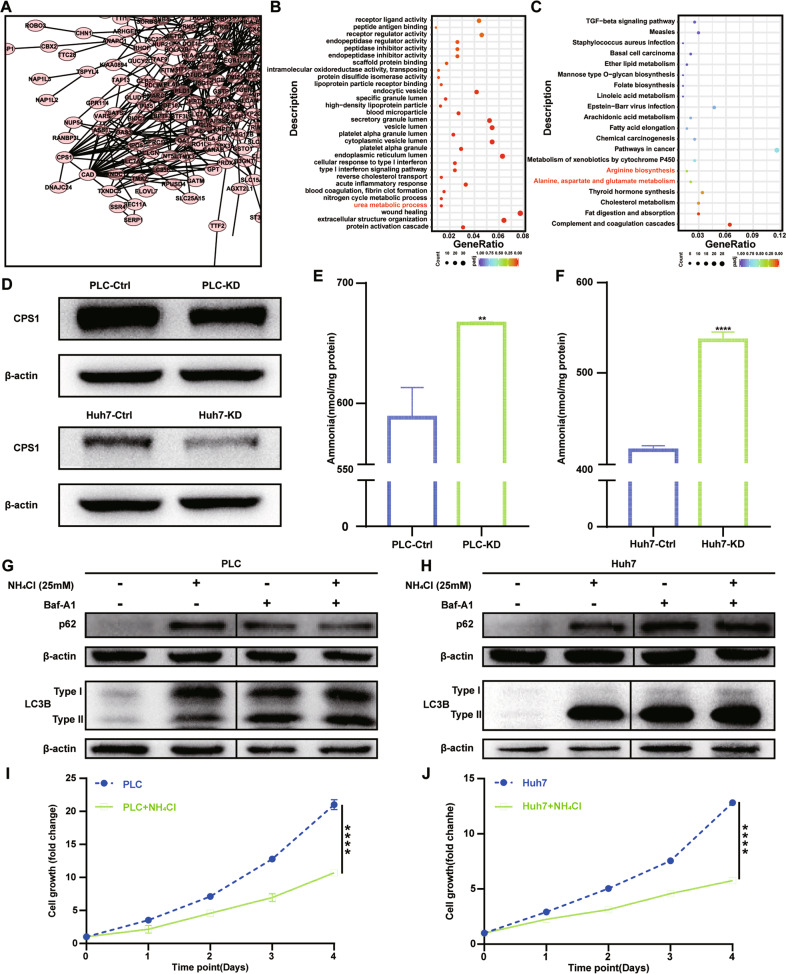
DNAJC24 knockdown inhibits HCC cells proliferation and autophagy by affecting the metabolism of ammonia. **A** Interactions in the STRING protein interaction database were used to analyze DEGs interaction networks. **B**, **C** GO functional enrichment analysis (**B**) and KEGG pathway enrichment analysis (**C**) of DEGs was performed to identify functionally related gene pathways. **D** Western blotting was performed to determine protein levels of CPS1. **E**, **F** NH_4_^+^/NH_3_ levels were measured in the PLC-KD (**E**) cells or Huh7-KD (**F**) cells as well as in the corresponding control cells using an ammonia assay kit by spectrophotometric analysis at 570 nm with extrapolation from the standard curve and expressed in nmol/mg protein. **G**, **H** NH_4_Cl (final concentration 25 mM) or/and Bafilomycin A1 (final concentration 400 nM) was added to the culture medium of PLC or Huh7 and incubated for 24h, respectively. Western blotting was performed to determine protein levels of LC3B. **I**, **J** CCK-8 cell viability assay analysis of the impact of NH_4_Cl (final concentration 25 mM) on PLC (**I**) and Huh7 (**J**) cell growth. Results were normalized to viability at day 0 and represented as fold change. Data were presented as mean ± SEM. *n* = 3–5. **P < 0.01, ****P < 0.0001.

**Fig. 7:**
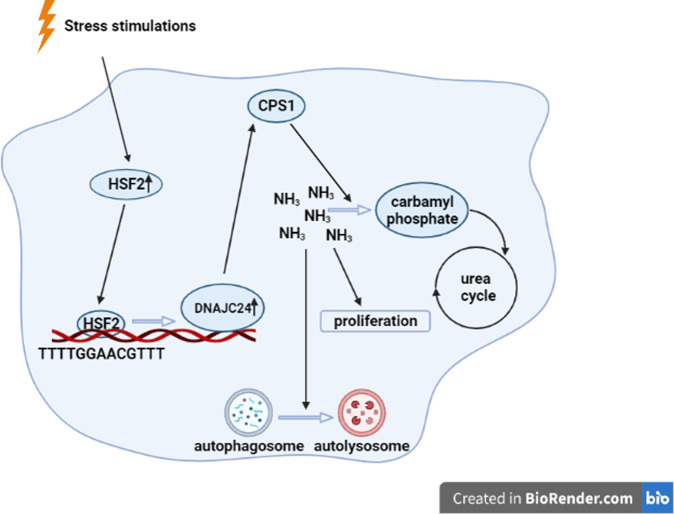
Schematic model of research conclusions. Extracellular stress could upregulate DNAJC24 expression through transcriptional regulation of HSF2 and targeting DNAJC24 can affect the autophagy and proliferation of HCC cells by decreasing the expression of CPS1, the first key enzyme of the urea cycle.

To further investigate how knocking down DNAJC24 affects the proliferation and autophagy of HCC cells, we performed whole-transcriptome sequencing on PLCDNAJC24-KD cells and control cells (Fig. S8A). As shown in the volcano plot, 323 genes were upregulated, and 183 genes were downregulated after DNAJC24 knockdown with the threshold of a *P* < 0.05 and a | log2 FC | > 0.0 (Fig. S8B). Based on the RNA-seq results, we applied the interactions in the STRING protein interaction database for the analysis of DEGs interaction networks. We identified two proteins with potentially relevant roles for DNAJC24: CPS1(Carbamoyl-phosphate synthase 1) and CAD (Carbamoyl-phosphate synthetase 2, aspartate transcarbamylase, and dihydroorotase) (Fig. 6A). Many metabolism-related pathways were enriched, such as cholesterol metabolism, amino acid metabolism, fatty acid metabolism by pathway enrichment analysis based on the DEGs (Fig. 6B, C). Notably, GO analysis suggested that DNAJC24 affected the urea metabolic process (Fig. 6B). In addition to pathways in cancer, KEGG pathway enrichment analysis showed that genes in the arginine biosynthesis and alanine, aspartate, glutamate metabolism pathways were enriched (Fig. 6C). CPS1 catalyzes the rate-limiting step of the urea cycle. Arginine, alanine, aspartate and glutamate are also closely associated with the urea cycle. This suggests that DNAJC24 knockdown affects the urea cycle. The further results showed that CPS1 expression was decreased in DNAJC24-KD cells (Fig. 6D). CPS1 initiates nitrogen disposal by catalyzing the production of carbamoyl phosphate (CP) from ammonia and bicarbonate in mitochondria. DNAJC24 knockdown resulted in intracellular ammonia accumulation (Fig. 6E, F). It has been reported that intracellular ammonia accumulation affects autophagy [38, 39] and proliferation [40]. Next, we examined the effect of ammonia on autophagy in PLC and Huh7 cells. NH_4_Cl (25 mM) significantly led to intracellular LC3B accumulation (Fig. 6G left and H left). We also observed that the addition of Baf-A1 caused this difference to disappear (Fig. 6G right and H right). The effect of NH_4_Cl on autophagy was identical to the effect of DNAJC24 knockdown on autophagy, suggesting that knocking down DNAJC24 affects autophagy by elevating intracellular ammonia. Finally, we assayed the effect of ammonia on the proliferation of PLC and Huh7 cells. CCK-8 cell viability assay confirmed that NH_4_Cl (25 mM) significantly inhibited the proliferation of PLC and Huh7 cells (Fig. 6I, J). In summary, our findings demonstrated that extracellular stress could upregulate DNAJC24 expression through transcriptional regulation of HSF2 and targeting DNAJC24 can affect the autophagy and proliferation of HCC cells by interfering with ammonia metabolism, thereby inhibiting the malignant development of HCC (Fig. 7).

## Discussion

The key findings of this report are as follows. First, extracellular stress can upregulate DNAJC24 expression through transcriptional regulation of HSF2, and high expression of DNAJC24 can promote proliferation and motility of HCC cells, leading to poor prognosis of HCC patients; second, we confirmed that targeting DNAJC24 can affect the proliferation and autophagy of HCC cells by interfering with ammonia metabolism, thereby inhibiting the malignant development of HCC.

HSPs are a large family of chaperones that are involved in protein folding and maturation of a variety of “client” proteins protecting them from degradation, oxidative stress, hypoxia, and thermal stress [41]. DNAJ/HSP40 is a large and diverse family with more than 40 different members. DNAJ/HSP40 family members have been documented to influence tumor progression by participating in the stress response [42, 43]. In contrast, whether DNAJC24 is involved in the stress response and its role in tumors hasn’t been studied. In this study we demonstrated that extracellular stress could upregulate DNAJC24 expression through transcriptional regulation of HSF2 and that high expression of DNAJC24 promotes proliferation and motility of HCC cells, leading to poor prognosis of HCC patients. By immunohistochemistry staining of 167 HCC tissues, we found that the expression of DNAJC24 correlated with tumor size and vessel density. As tumor size increases, there are various stresses within the tumor, and increased vascular density is a coping strategy for hypoxia [44] and nutrient deficiency [45, 46]. DNAJC24 may bridge the gap between tumor size and vascular density.

HSFs were originally described to recognize a consensus heat shock element (HSE) DNA binding site and activate genes encoding protein chaperones in response to elevated temperatures [27]. Whether HSFs can regulate DNAJC24 expression or which member regulates DNAJC24 expression has not been reported. In our study, we found that HSF2 was highly correlated with DNAJC24, but not HSF1. Data from TCGA database also confirm that HSF2 shares the same expression pattern as DNAJC24 in HCC, such as higher expression in cancerous tissues and higher expression in advanced HCC. Moreover, the expression levels of both HSF2 and DNAJC24 are increased by the application of external stimuli. Most importantly, high expression of HSF2 is also associated with poor prognosis in HCC patients. We eventually confirmed that HSF2 can regulate DNAJC24 expression by a dual-luciferase reporter assay. HSF1 can promote cancer initiation and progression, which has been proven by numerous studies [27, 47]. Compared to HSF1, the role of HSF2 in malignancy is inconclusive. J K Björk et al. demonstrated that HSF2 is a suppressor of prostate cancer invasion [48], but another study demonstrated that HSF2 was increased in lung cancer and associated with the occurrence of lung cancer by enhancing the expression of HSPs [49]. However, our study showed that HSF2 is a cancer-promoting gene in HCC. We also note that activation of HSF1 is a multistep and highly regulated process. Under normal conditions, mammalian HSF1 exists mainly in an inactive form. In response to various stresses HSF1 is converted to an active form with DNA binding capacity [50]. Thus, the transcriptional regulatory effect of HSF1 on target genes is dependent on activity changes rather than expression. Therefore, the absence of correlation between HSF1 and DNAJC24 doesn’t deny that HSF1 may transcriptionally regulate the expression of DNAJC24. Whether HSF1 can regulate DNAJC24 needs to be further investigated.

Under normal culture conditions, DNAJC24 knockdown affected neither the cell cycle nor apoptosis of HCC cells but did affect autophagy. Autophagy is a highly regulated catabolic process, involved in the turnover of damaged organelles and cytoplasmic material to fuel starving cells and to maintain cellular homeostasis during either normal or stress conditions [51]. Therefore, autophagy is also a strategy for cells to cope with stress. In this respect, knocking down DNAJC24 did affect the cellular response to stress. RNA-seq revealed that DNAJC24 knockdown affected CPS1 and thus ammonia metabolism. CPS1 is a mitochondrial enzyme that catalyzes the first committed step of the urea cycle, which is important for the removal of excess ammonia from the cells. It has been demonstrated that reduced CPS1 expression leads to intracellular ammonia accumulation [40, 52]. Le Li et al. demonstrated that reduced CPS1 expression leads to intracellular ammonia accumulation and thus inhibits cell proliferation [40]. Our study confirmed for the first time that DNAJC24 can affect autophagy in hepatocellular carcinoma by interfering with ammonia metabolism.

## Supplementary information

Supplementary Fig 1

Supplementary Fig 2

Supplementary Fig 3

Supplementary Fig 4

Supplementary Fig 5

supplementary Figure 6

supplementary Figure 7

supplementary Figure 8

Supplementary Figure Legends

Supplementary Table 1

Supplementary Table 2

Supplementary Table 3

authorship contribution statement

reproducibility checklist

Full length western blots

Original Data File

## Data Availability

All data are fully available without restrictions.
