# Supplementary figures and images for: DNAJC24 is a potential therapeutic target in hepatocellular carcinoma through affecting ammonia metabolism
Source: Cell Death Dis. 2022 May 24;13(5):490. doi: 10.1038/s41419-022-04953-z (PMC9127113; doi:10.1038/s41419-022-04953-z)

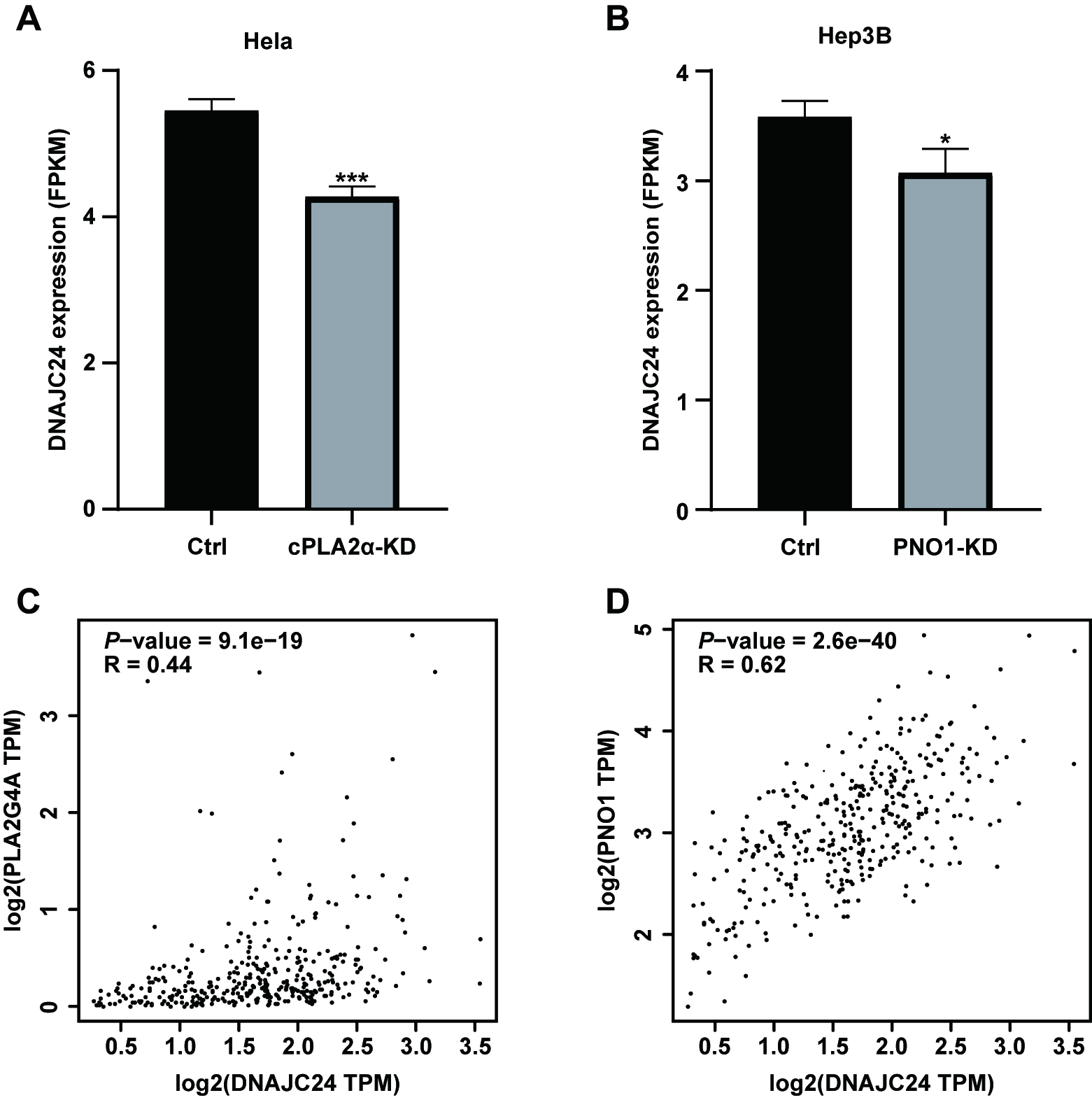

Supplement: Supplementary file 1 — Supplementary Fig 1 [file 41419_2022_4953_MOESM1_ESM.tif]

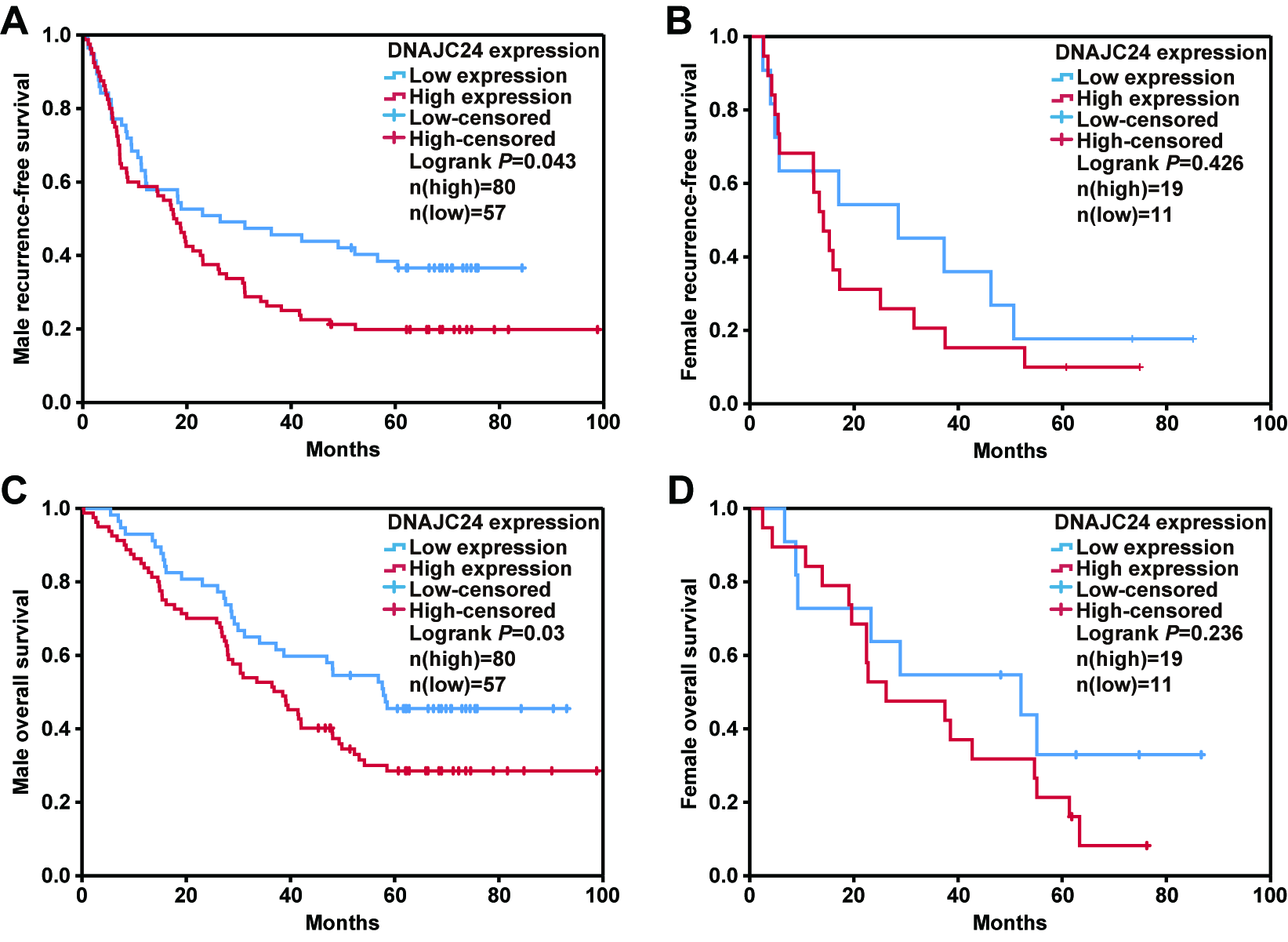

Supplement: Supplementary file 2 — Supplementary Fig 2 [file 41419_2022_4953_MOESM2_ESM.tif]

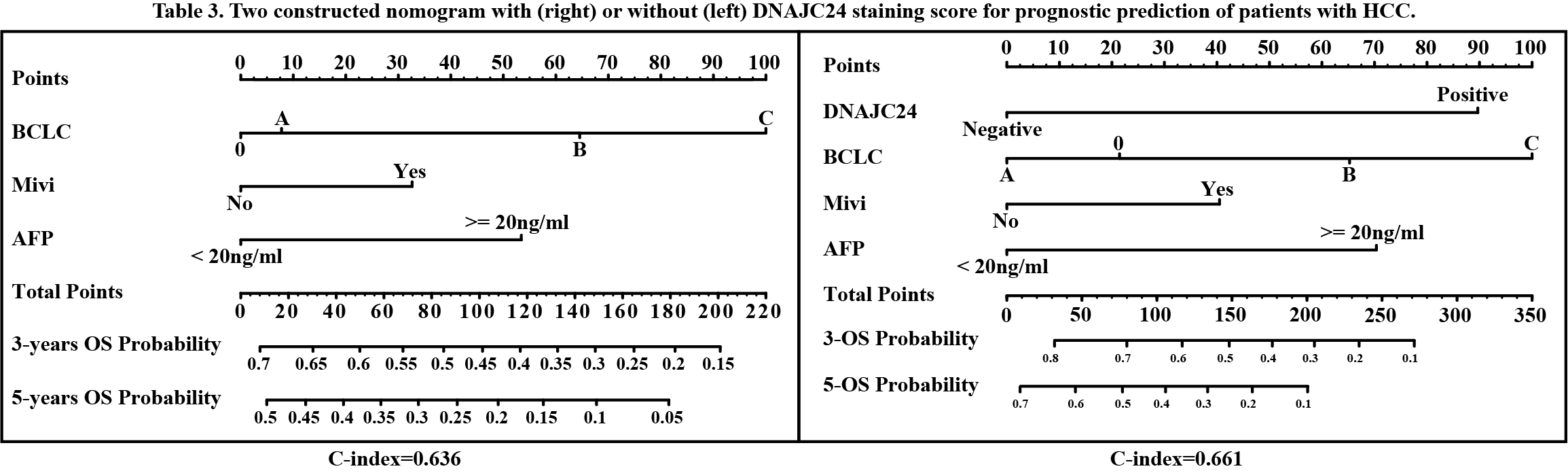

Supplement: Supplementary file 3 — Supplementary Fig 3 [file 41419_2022_4953_MOESM3_ESM.tif]

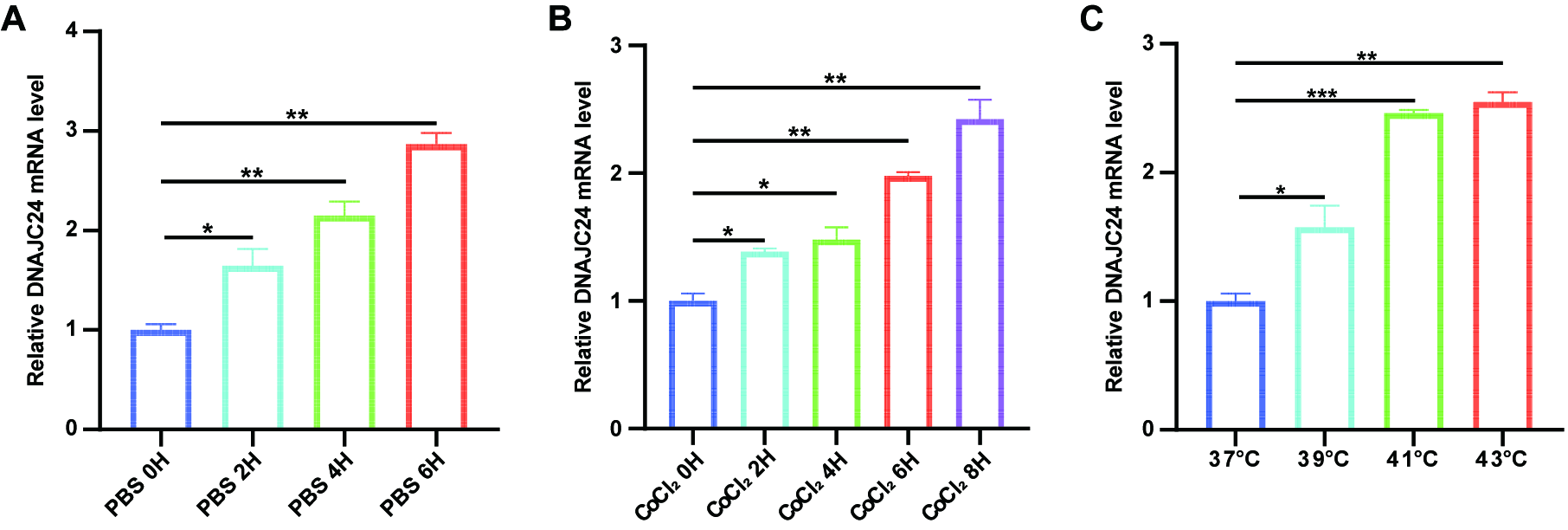

Supplement: Supplementary file 4 — Supplementary Fig 4 [file 41419_2022_4953_MOESM4_ESM.tif]

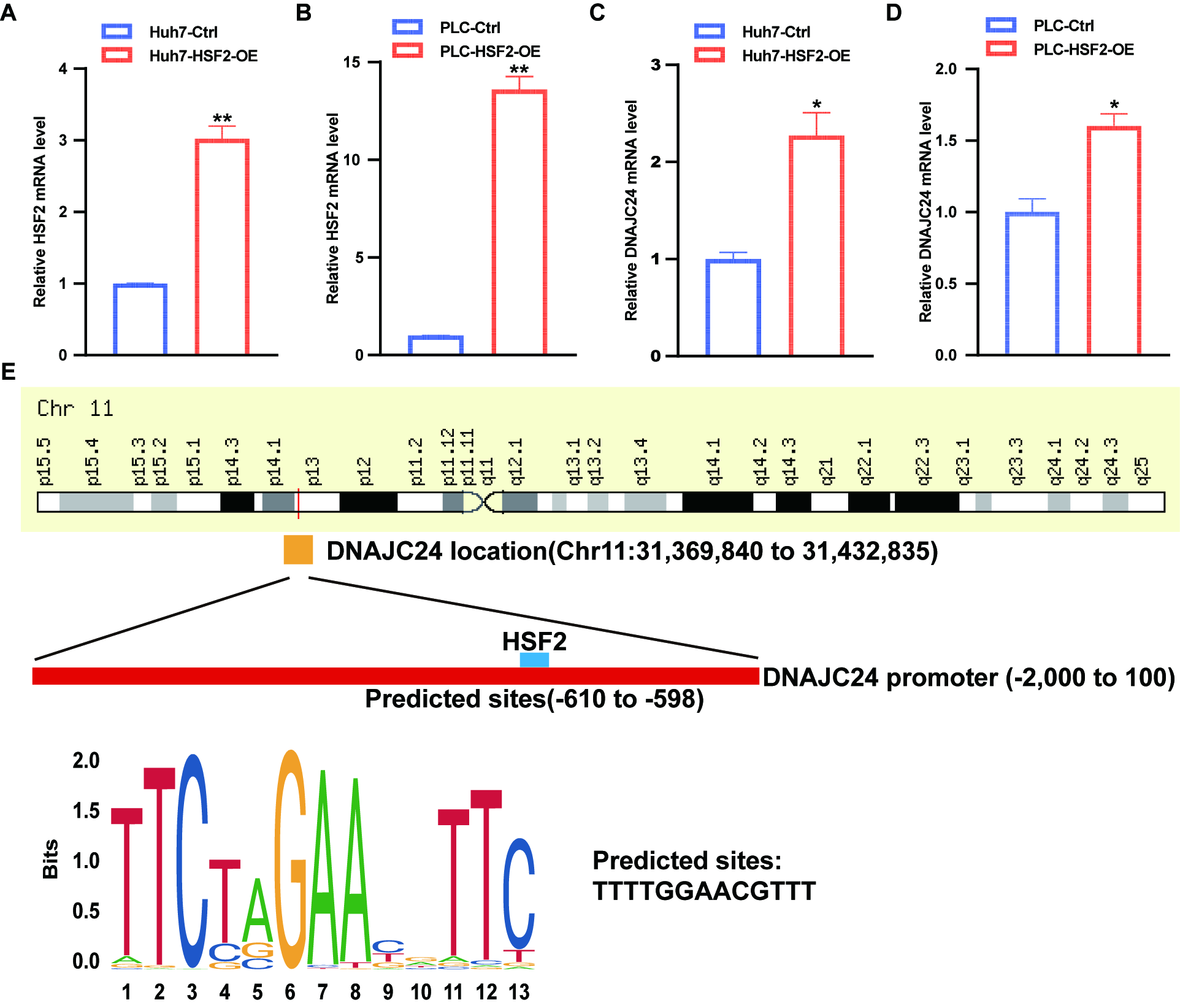

Supplement: Supplementary file 5 — Supplementary Fig 5 [file 41419_2022_4953_MOESM5_ESM.tif]

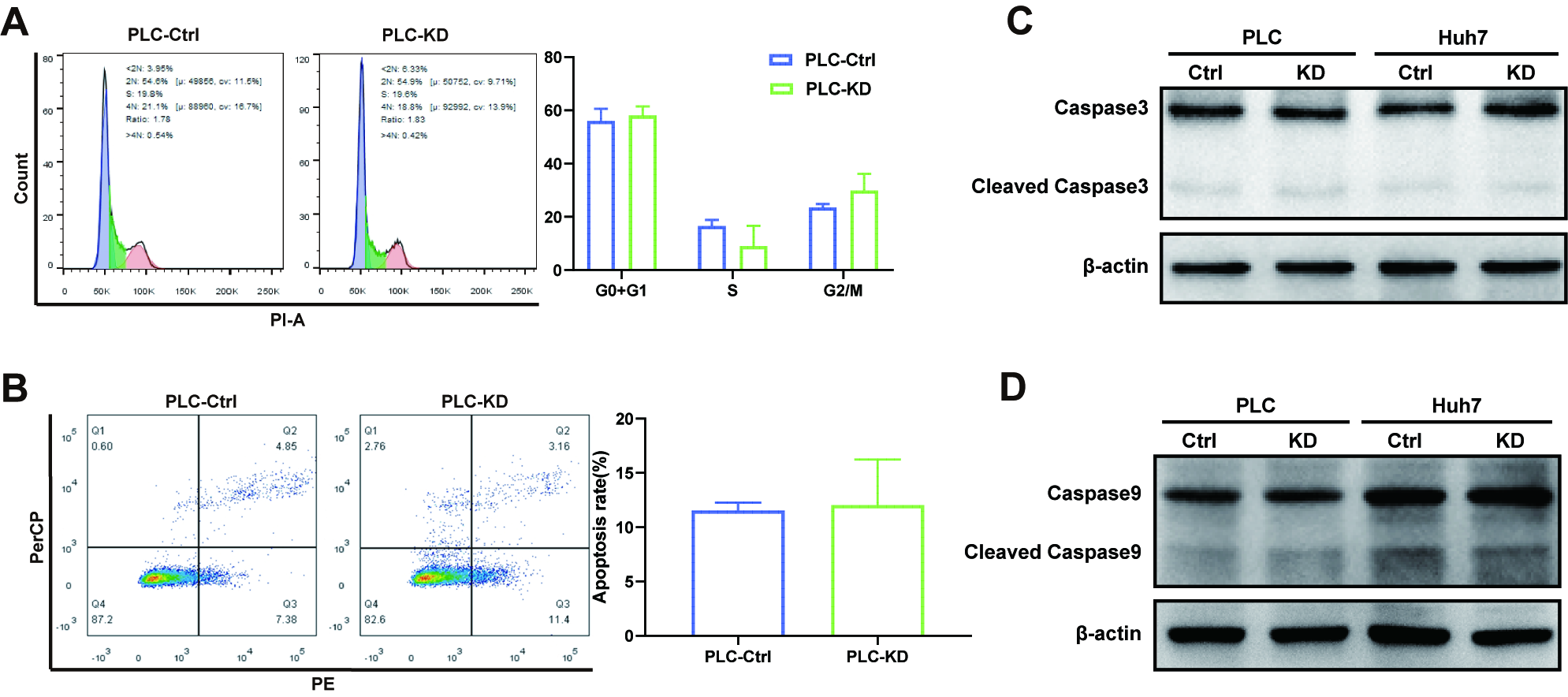

Supplement: Supplementary file 6 — supplementary Figure 6 [file 41419_2022_4953_MOESM6_ESM.tif]

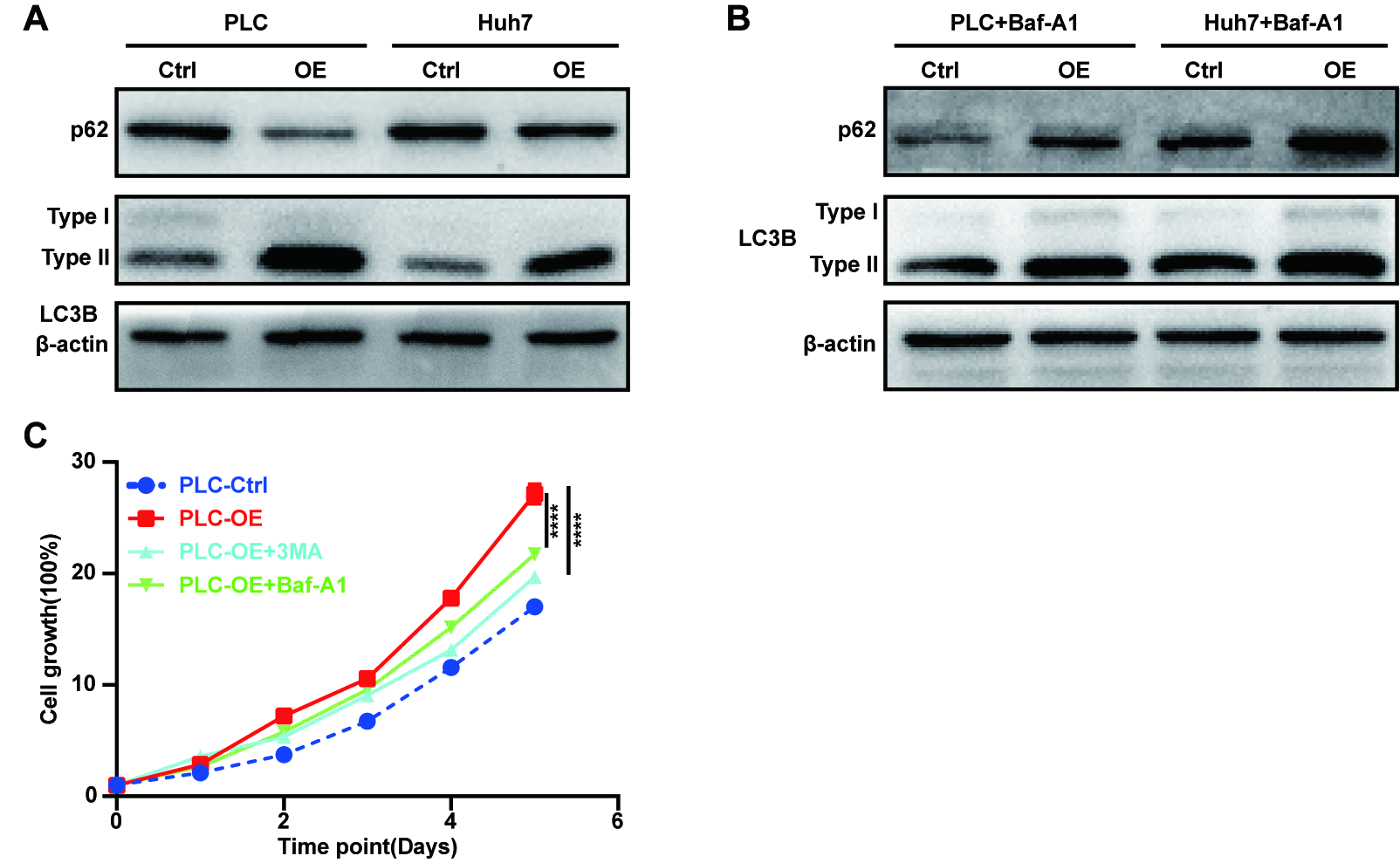

Supplement: Supplementary file 7 — supplementary Figure 7 [file 41419_2022_4953_MOESM7_ESM.tif]

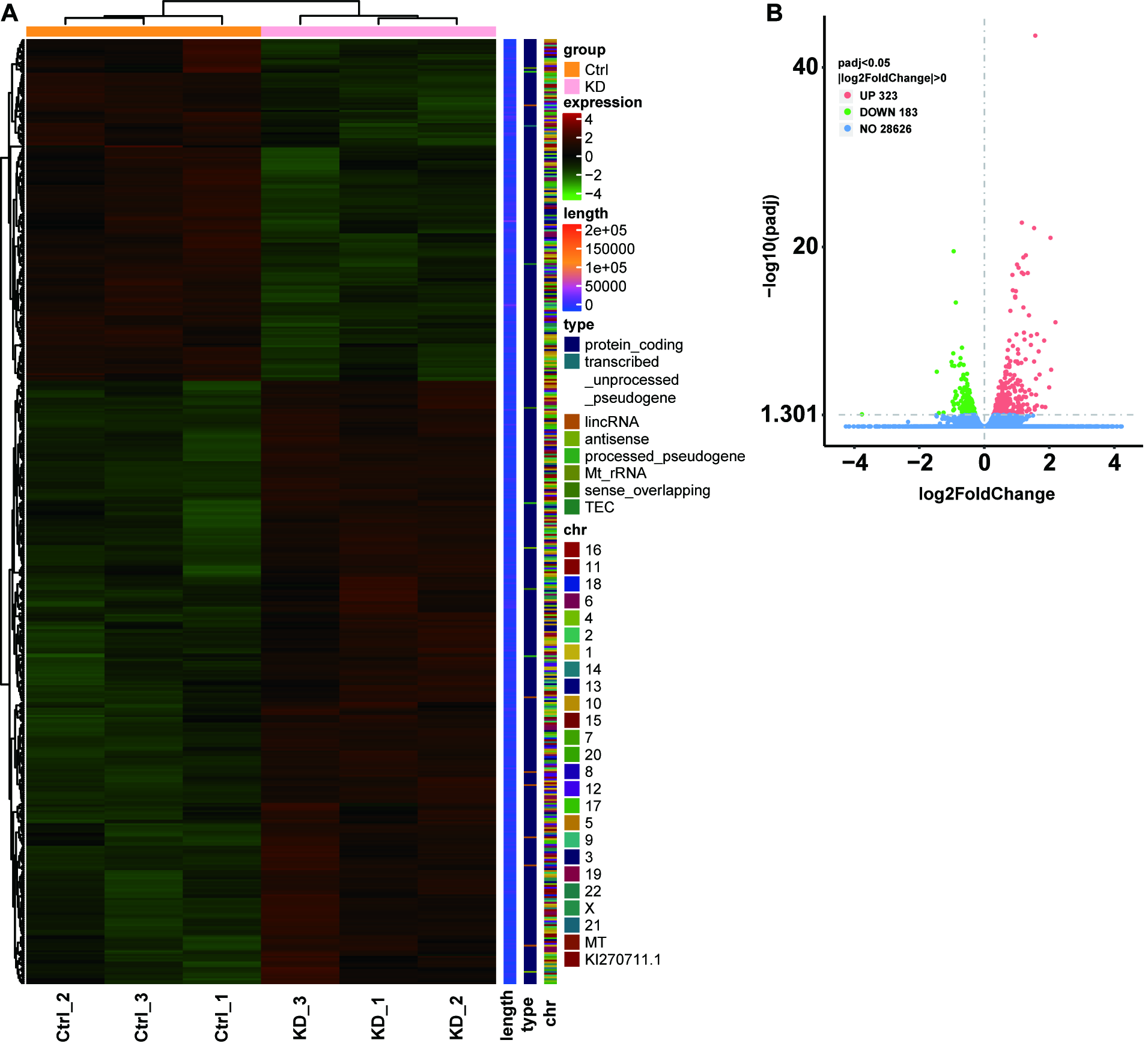

Supplement: Supplementary file 8 — supplementary Figure 8 [file 41419_2022_4953_MOESM8_ESM.tif]
