# Supplementary material for: DNAJC24 is a potential therapeutic target in hepatocellular carcinoma through affecting ammonia metabolism
Source: Cell Death Dis. 2022 May 24;13(5):490. doi: 10.1038/s41419-022-04953-z (PMC9127113; doi:10.1038/s41419-022-04953-z)
Supplement: Supplementary file 9 — Supplementary Figure Legends [file 41419_2022_4953_MOESM9_ESM.docx]

**Supplementary Figure Legends**

**Supplementary Figure 1.** DNAJC24 was positively correlated with cPLA2α and PNO1at the mRNA level. **A, B** Transcriptome sequencing revealed that DNAJC24 expression was downregulated in cells with cPLA2α (**A**) or PNO1 (**B**) knockdown. **C, D** A positive correlation between DNAJC24 and cPLA2α (**C**), PNO1 (**D**) at the mRNA level in HCC according to the GEPIA database. Data were presented as mean ± SEM. n= 3. **P* < 0.05, ****P* < 0.001.

**Supplementary Figure 2.** Survival analysis after grouping 167 patients by gender. **A, C** The Kaplan–Meier survival analysis of recurrence-free survival (**A**) and overall survival (**C**) in male HCC patients. **B, D** The Kaplan–Meier survival analysis of recurrence-free survival (**B**) and overall survival (**D**) in male HCC patients.

**Supplementary Figure 3.** Two constructed nomogram without (left) or with (right) DNAJC24 staining score for prognostic prediction of patients with HCC.

**Supplementary Figure 4.** The effects of starvation (**A**), hypoxia (**B**) and heat stimulation (**C**) on the expression level of DNAJC24 in PLC cells were detected by qRT-PCR. Data were presented as mean ± SEM. n= 2. **P* < 0.05, ***P* < 0.01, ****P* < 0.001.

**Supplementary Figure 5. A-D** PLC and Huh7 cells were infected with a lentivirus to produce stable HSF2 overexpression (HSF2-OE) cells. qRT-PCR was performed to determine levels of HSF2 (**A, B**) and DNAJC24 (**C, D**) mRNA. β-actin was used as an internal control. **E** JASPAR website shown that HSF2 may bind to a segment of the DNAJC24 promoter sequence(-TTTTGGAACGTTT-). Data were presented as mean ± SEM. n= 2. **P* < 0.05, ***P* < 0.01.

**Supplementary Figure 6. A** Cell cycle assay revealed that DNAJC24 knockdown didn't result in significant changes in the cell cycle of PLC cells under normal culture conditions. **B** The apoptosis rate of PLC DNAJC24-KD and control cells was measured by Annexin V/PI double staining under normal culture conditions. No significant differences were found. **C** Detecting caspase 3 and cleaved caspases3 in DNAJC24-KD cells as well as in control cells by Western blotting. **D** Detecting caspase 9 and cleaved caspases9 in DNAJC24-KD cells as well as in control cells by Western blotting. Data were presented as mean ± SEM. n= 3.

**Supplementary Figure 7. A, B** DNAJC24-OE cells and control cells were cultured under normal conditions (**A**) or in medium supplemented with Baf-A1 (**B**, final concentration 400nM) for 24H. Western blotting was performed to determine protein levels of LC3B and p62 in cell lysates. **C** CCK-8 cell viability assay was performed to analyze the effect of 3MA (final concentration 5mM) and Baf-A1 (final concentration 400nM) on the growth of PLC-DNAJC24-OE cells. Results were normalized to viability at day 0 and represented as fold change. Data were presented as mean ± SEM. n= 5. *****P*< 0.0001.

**Supplementary Figure 8.** Heat map (**A**) and volcano map (**B**) showing the DEGs between DNAJC24-KD cells and control cells.
