# Supplementary material for: DNAJC24 is a potential therapeutic target in hepatocellular carcinoma through affecting ammonia metabolism
Source: Cell Death Dis. 2022 May 24;13(5):490. doi: 10.1038/s41419-022-04953-z (PMC9127113; doi:10.1038/s41419-022-04953-z)
Supplement: Supplementary file 13 — authorship contribution statement [file 41419_2022_4953_MOESM13_ESM.docx]

CRediT authorship contribution statement

**Guangtao Li:** Investigation, Formal analysis, Data curation, Writing original draft. **Yuchao He:** Investigation, Formal analysis, Data curation. **Hui Liu:** Investigation, Formal analysis, Data curation. **Dongming Liu:** Investigation, Formal analysis, Data curation. **Lu Chen:** Investigation, Formal analysis, Data curation. **Yi Luo:** Conceptualization, Methodology, Formal analysis, Data curation. **Liwei Chen:** Conceptualization, Methodology, Formal analysis, Data curation. **Lisha Qi:** Investigation, Data curation, Formal analysis. **Yun Wang:** Investigation, Data curation, Formal analysis. **Yingying Wang:** Investigation, Data curation, Formal analysis. **Yu Wang:** Investigation, Data curation, Formal analysis. **Linlin Zhan:** Data curation, Formal analysis. **Ning Zhang:** Conceptualization, Methodology, Formal analysis, Data curation. **Xiaolin Zhu:** Conceptualization, Supervision, Formal analysis, Data curation, Writing review & editing. **Tianqiang Song:** Conceptualization, Supervision, Formal analysis, Data curation, Writing review & editing. **Hua Guo:** Conceptualization, Supervision, Formal analysis, Data curation, Writing review & editing
